# Mechanisms of Sports Concussion in Taekwondo: A Systematic Video Analysis of Seven Cases

**DOI:** 10.3390/ijerph191610312

**Published:** 2022-08-19

**Authors:** Sunghe Ha, Min Jin Kim, Hee Seong Jeong, Inje Lee, Sae Yong Lee

**Affiliations:** 1 Department of Physical Education, Yonsei University, Seoul 03722, Korea; 2 International Olympic Committee Research Centre KOREA, Seoul 03722, Korea; 3 Department of Sports and Health Management, Mokwon University, Daejeon 35349, Korea; 4 Department of Sports Rehabilitation Medicine, Kyungil University, Gyeongsan 38428, Korea; 5 Institute of Convergence Science, Yonsei University, Seoul 03722, Korea

**Keywords:** combat sports, competition, concussion, head blow, head injury, martial arts

## Abstract

Sports-related traumatic brain injuries are the most common injury in adolescents and young adults due to recurrent concussion experiences and head shock. Therefore, this study was designed to describe player characteristics and situational factors associated with concussions in the World Taekwondo Championships using systematic video analysis. Athlete injury data were collected using a web-based injury surveillance system at the World Taekwondo Championships organized by World Taekwondo from 2017 to 2019. Seven video footage were independently analyzed by four analysts using a modified Heads-Up Checklist. Descriptive statistical analysis was used. The incidence of concussion was 3.21 per 1000 games. Most players with concussions were shorter than their opponents, and most concussions were caused by a roundhouse kick on the front of the face. Regarding the acceleration direction of the head after the impact, transverse and multiplane directions were the most common. Most players with a concussion have used a closed stance and did not use blocking techniques during the defense. The rate of concussions caused by penalties was 42.9%. Based on our findings, no other injury mechanisms, except for direct blows to the head, were observed. Therefore, education on the risk and symptoms of concussion, the appropriate management and blocking techniques should be emphasized in TKD-S to reduce incidence of concussion.

## 1. Introduction

Taekwondo sparring (TKD-S) is an official Olympic sport and is scored by kicking and punching the head and body: one point for a body punch, two points for a body kick, three points for a head kick, four points for a body turning kick and five for a head turning kick [1]. In Taekwondo, the head kick is the most frequent attack skill because it has the highest score [2]. Given the scoring system in TKD-S, as a turning kick is more valuable than other kicks, it is used frequently. However, it can make a greater angular momentum. Therefore, the magnitude of impact of a turning kick to the trunk and head may cause brain damage.

Sports-related concussion is defined as a traumatic brain injury caused by a direct impact on the head, face and neck or by a biomechanical force applied to the body that transmits the impact force to the head [3]. Due to the nature of TKD-S, athletes tend to be more exposed to concussions, and the incidence rate (IR) ranges from 0 to 50.2 per 1000 athlete exposures (AEs) (median: 4.9), which is less than that in boxing but higher than that in other contact sports [4]. Considering the adverse effects of concussion history on athletes, understanding not only the mechanism of concussion caused by head attacks but also the possible mechanism of concussion in Taekwondo is crucial.

Since head attacks are used to win a score, protective equipment must be worn to participate in TKD-S, including headgear, body protection and mouth guard [1]. In other contact sports, the risk of concussion was reduced by 43–57% and 80% by wearing a headgear and a mouth guard, respectively [5]. However, concussions are frequent in TKD-S, even though TKD-S athletes wear the aforementioned protective equipment. Therefore, identifying how concussions occur under the protected condition by equipment in TKD-S is essential.

To understand the mechanism of sports-related concussion, systematic video analysis is useful. In ice hockey and rugby, systematic video analysis is being extensively used to understand the mechanism of concussion [6,7,8]. In previous video analysis studies of TKD-S, the cause of concussion was the direct blow to the head [9,10]. In addition to head blows, understanding other possible mechanisms is necessary to reduce the IR of concussion.

Sports-related traumatic brain injuries are the most common injury in adolescents and young adults due to recurrent concussion experiences and head shock, accounting for 1.2–30.3% of all traumatic brain injury occurrences [11]. It is also reported as a risk factor for mild cognitive impairment among retired athletes [12,13,14]. Among elite athletes who have participated in the sport from an early age, if they understand the mechanisms of concussion, they will be able to cope properly with injury occurrence. Therefore, this study was designed to describe player characteristics and situational factors associated with concussions in the World Taekwondo Championships using systematic video analysis. The hypothesis of this study is that the cause of concussion may be caused not only by head kicks but also by other shock mechanisms.

## 2. Materials and Methods

### 2.1. Overview

Athlete injury data were collected using a web-based injury surveillance system at the World Taekwondo Championships organized by World Taekwondo (WT) from 2017 to 2019. Based on the injury data officially reported by WT, the concussion event footage in the video database system was analyzed. In 3 years, 10 concussions were reported in five major competitions, which comprised seven adult cases and three junior cases. This secondary analysis study used injury surveillance and video data of World Championships hosted by World Taekwondo. This study was conducted in accordance with the guidelines of the declaration of Helsinki and was approved by the institutional review board of Yonsei University.

### 2.2. Concussion Data Collection

Depending on the scale of the major event, 5–10 medical doctors (MDs), 10–20 medical staff and 3–6 injury surveillance staff were pretrained and assigned to the competition site. All injury data were recorded in the web-based injury surveillance and video database system of WT.

Concussion data during matches were extracted from the injury data of 3223 (i.e., 1857 males and 1366 females) athletes. All injuries were diagnosed by the MDs. In this study, systematic video analysis was performed on 10 cases: seven concussions in 1361 senior males and 973 senior females and three concussions in 496 junior males and 393 junior females. The age criterion was 17 years or older for seniors and 15–17 years for juniors [1]. Weight classes were classified according to body weight, which were different according to competition characteristics [1].

### 2.3. Concussion Definition

Taekwondo concussion is designed as a traumatic brain injury induced by biomechanical forces [15]. According to WT, Taekwondo concussion during the competition was defined as follows: (1) There is no loss of consciousness after a head injury; however, the balance is broken or it takes time to get up due to shock, and the referee could not resume the game while counting 10. Medical opinion on a concussion from an MD is submitted during Sport Concussion Assessment Tool 5 (SCAT5) examination in the infirmary (short memory and impaired balance, among others). (2) There is a loss of consciousness for more than a few seconds to less than 1 min due to a brief knockout after a head injury, and the referee could not resume the game while counting 10. (3) There is a head injury, and the athlete falls to the floor and could not get up immediately; the referee stops the game and immediately calls a doctor to check the athlete on the mat; and the athlete then wakes up within 1 min but shows incomplete recovery. However, if the injured athlete could not communicate with the medical staff or the doctor is not familiar with the SCAT5, the concussion was diagnosed using questions on basic symptoms, including headache, dizziness, nausea/vomiting, cloudiness and blurry vision, which are suspected in a concussion during physical examination [15].

### 2.4. Video Analysis

**Figure 1:**
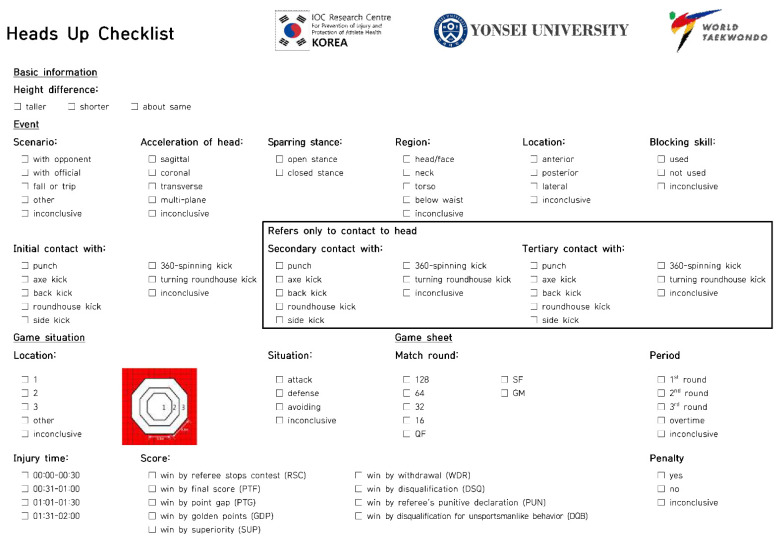
The modified Heads-Up Checklist.

**Figure 2:**
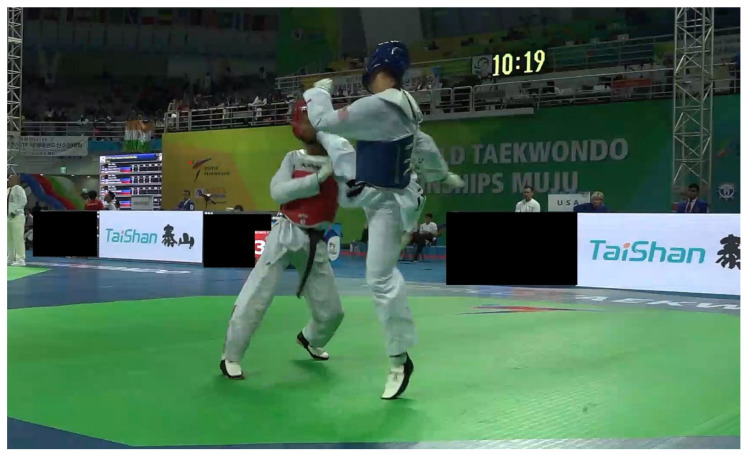
A case of head blow during Taekwondo sparring in this study.

Seven video footages were independently analyzed by four analysts using a modified Heads-Up Checklist (mHUC) (Figure 1 and Figure 2) [10,16]. The mHUC consisted of 16 components, representing the situation of concussions (Figure 1). Systematic video analysis was performed using Kinovea 0.8.27 [17]. The analysts solved disagreements by discussing the issues until a consensus was reached after video analyses.

The inter-rater reliability of systematic video analysis was determined by calculating Fleiss’ kappa using R statistical language and RStudio (version 1.0.143, RStudio, Inc., Boston, MA, USA). Fleiss’ kappa was used to calculate the multi-rater kappa for the inter-rater reliability [18]. According to the guidelines proposed by Landis and Koch [19], the kappa coefficient was interpreted as follows: <0.00 = poor reliability; 0.00–0.20 = slight reliability; 0.21–0.40 = fair reliability; 0.41–0.60 = moderate reliability; 0.61–0.80 = substantial agreement; and 0.81–1.00 = almost perfect agreement [19]. All analysts exhibited almost perfect reliability with 0.90 of Fleiss’ kappa (*p* < 0.001).

### 2.5. Statistical Analysis

The outcome variables on concussions during TKD-S were assessed using descriptive statistics. The IR of concussion in TKD-S was calculated using the following formula [20].

IR = # of total concussion cases/# of total AEs × 1000(1)

The IR of competition was expressed as the number of injuries per 1000 Taekwondo athlete games (AEs) and per 1000 athlete game minutes (mins) [11]. All statistical analyses were performed using Statistical Package for the Social Sciences (version 24.0; IBM Corp., Armonk, NY, USA) and were considered statistically significant if two-tailed *p* values are less than 0.05.

## 3. Results

### 3.1. Incidence Rate of Concussion

Ten concussions occurred in the World Taekwondo Championships organized by WT from 2017 to 2019 (3.21/1000 AEs; 0.63/1000 min). Of the 10 concussions, 7 (70%) were identified on video footages and analyzed in this study. Of the three excluded events from the 10 concussions, one was excluded because the image was lost, and the remaining two events were excluded because distinguishing the concussion event on the images was impossible.

### 3.2. Situation of Concussion

Of the seven players with concussions, four were shorter than their opponents, two had approximately the same height as their opponents, and one was taller height than their opponent (Table 1). All concussions were caused by contact with an opponent (Table 1). All impact regions that caused concussion were faces, and the most common areas were in the anterior of the face, followed by the lateral part of the face (Table 1). Regarding the direction of the acceleration of the head after the impact, transverse and multiplane directions were the most common, followed by the sagittal plane direction (Table 1).

In the initial contact, concussion was most frequently caused by the opponent’s roundhouse kick, followed by the opponent’s punch (Table 1). Secondary contact with the mat was observed after the initial contact, while ‘not applicable’ was observed in more than half of the cases (Table 1). No tertiary contact was observed. When the concussion occurred, for the sparring stance, the closed stance was most frequently observed, followed by the open stance (Table 1). Moreover, the players with a concussion were shown in the stand, and the other was the forward step (Table 1).

One case of concussion occurred while the player was attacking, and the others occurred during defense (Table 1). During the occurrence of concussion, six cases did not use blocking techniques, and the other case failed to defend with a foul attack (Table 1). Three penalties were observed, and the others had no penalty (Table 1). Regarding the period of concussion occurrence, concussions occurred most frequently in the third round of the match, followed by the first and second rounds (Table 1). The injury time of each round was most frequently observed at 01:31–02:00, followed by 00:31–01:00, 00:00–00:30 and 01:01–01:30 (Table 1). As for the location of occurrence in the court, area 2 was the most common, followed by area 3 (Table 1).

## 4. Discussion

This study was designed to describe player characteristics and situational factors associated with concussions in the WT Championships using systematic video analysis.

### 4.1. Bias in Estimating the Incidence of Concussion

In this study, the incidence of concussions was 3.21 per 1000 games. However, the incidence of concussions in a previous systematic review was 13.8/1000 AEs for men and 12.1/1000 AEs for women, who were Taekwondo athletes, which were higher than that found in this study [11]. Unlike previous studies, this study included the competitions from 2017 to 2019 since the introduction of the electronic protective gear in 2010. This difference may have caused the athletes to not need strong blows as the scoring system scores each blow using the sensor attached to the protective gear. Furthermore, the medical staff who diagnosed concussions had to watched multiple matches, not just the matches in the competition. Oler et al. have reported 2/1000 AEs in a situation when a physician had to watch up to 10 games, which is thought to be the reason for the low incidence of concussion under similar conditions as in this study [21]. Furthermore, concussion awareness among athletes remains low [22]. Given this tendency, an injured athlete tends to rarely come to the medical room to report or receive a diagnosis unless they have serious concussion symptoms, such as loss of consciousness. This is because when athletes are diagnosed with concussion by an MD, they are unable to participate in the remaining matches of the competition.

### 4.2. Low-Quality Blocking Technique

In this study, it was observed that the athletes diagnosed with concussion suffered a loss of consciousness or fall due to a direct blow to the face. The mechanism of Taekwondo concussion was reported as a direct blow to the head. The findings of Koh and Cassidy support our findings [23]. According to a previous study, the use of the upper extremity to protect the head and neck transmits an extreme shock causing a fracture [24]. A low-quality blocking technique was identified as the main cause of injuries in Taekwondo [2,25]. According to our results on height difference, shorter athletes suffered from concussion more than taller athletes who have an advantage to kick their opponent’s head. Therefore, relatively shorter athletes should develop blocking techniques in addition to avoiding skills, but 85.7% of the athletes failed to use blocking techniques, which may have led to a concussion.

In our study, most concussions occurred with standing or forward step in the edge of the court. Space for movement is limited in the edge of the court, which may indicate that an athlete cannot move backwards [1]. Additionally, standing or forward step may cause greater impulsive forces than backward step during impact. Therefore, education for using blocking techniques should be conducted for the aforementioned situation to reduce concussion. In an effort to lower the incidence of concussion in TKD-S, understanding the characteristics of thin and light headguards and using them along with blocking techniques can be effective in buffering [26]. Furthermore, it is thought that further training of avoidance skills for defense can significantly reduce the force applied to the head. The two cases not included in the analysis of this study were reported after the first match of the competition, and the mechanisms were unclear. When blocking techniques are not used, the shock is applied to the body without buffering. Therefore, frequent damages through a strong attack may cause concussions. Since concussions may or may not result in loss of consciousness, the amount of shock accumulated to the brain during the games should be observed along with the amount of shock enough to cause a concussion to diagnose concussion in future studies.

### 4.3. Protective Equipment: Headguards

A concussion would be effectively assessed through combined observation of linear and rotational acceleration [27]. In this study, it was observed that the opponent athlete most frequently kicked the front of the head of the injured athlete during the match, followed by the side of the head, resulting in a concussion as the head rotates in the transverse plane or multiplane. According to a previous study, the location of the impact on the head during TKD-S was mainly the side of the head, followed by the back and front [28]. Our findings were consistent with those of a previous study if excluding two penalties by punches to the face. In Taekwondo, protective equipment is used to prevent injuries; however, preventing concussions caused by axial rotation will be difficult because the rotation acceleration of the head cannot be controlled only by using headgears. Furthermore, it is suggested that wearing headguards cannot act as a protective device in preventing concussion with axial rotation as they induce greater torque by increasing the moment arm between the axis of rotation and the impact site because of thickness of a headguard.

### 4.4. Concussion Preventive Education

Due to the characteristics of TKD-S, preventing concussions through protective equipment may be difficult. Therefore, the risk of concussion should be educated to athletes, coaching staff and organizing committee, and if rules are not complied, strong punishment should be imposed. We found that two of three penalties caused by rule violation were punches to faces. In TKD-S, a player who gets a penalty with a punch to opponent’s head is just marked down [1]. In boxing, if a concussion occurs, participation in competitions is prohibited from 30 to 60 days depending on the degree of damage [29]. Even these days, when concussions have been raised as international public health issues, awareness and understanding of concussions were reported to be low [26]. Resuming exercise without understanding and proper management of concussions not only increases the risk of re-injury but also causes problems, such as traumatic brain injury [19]. If the repeated concussions are caused by a lack of understanding of concussions, concussion preventive education may improve this situation.

This study has a limitation, which may have limited the generalization of our findings. This study only involved a small sample size. However, it is considered as a valuable study to understand the concussion mechanisms in TKD-S. Furthermore, it is thought that our findings can suggest the next step to prevent concussions in TKD-S and the direction of future research in the sports field.

## 5. Conclusions

Through systematic video analysis, no other injury mechanisms, except for direct blows to the head, were observed. Since concussion caused by major mechanisms found in this study may not be prevented using protective equipment, such as headguards, education on the risk and symptoms of concussion and the appropriate managements is necessary to prevent sequelae of concussion. In addition, blocking techniques should be emphasized in TKD-S to reduce incidence of concussion.

## Figures and Tables

**Table 1:** Frequency distribution and percentage of variables for concussion events during Taekwondo sparring in the concussed players.

Variables	*n* (%)	Variables	*n* (%)	Variables	*n* (%)
Height Difference		Penalty (in Opponents)		Blocking Skill	
Taller	1 (14.3)	Yes	3 (42.9)	Used	0 (0)
Shorter	4 (57.1)	No	4 (57.1)	Not used	6 (85.7)
About same	2 (28.6)			N/A	1 (14.3)
Scenario		Initial contact with		Situation	
With opponent	7 (100.0)	Punch	2 (28.6)	Attack	1 (14.3)
Fall or trip	0 (0)	Axe kick	0 (0)	Defense	6 (85.7)
Other	0 (0)	Back kick	0 (0)	Avoiding	0 (0)
		Roundhouse kick	5 (71.4)		
		Side kick	0 (0)		
		360-spinning kick	0 (0)		
		Turning roundhouse kick	0 (0)		
		Mat	0 (0)		
Region		Secondary contact with		Period	
Head	0 (0)	Punch	0 (0)	1st round	2 (28.6)
Face	7 (100.0)	Axe kick	0 (0)	2nd round	1 (14.3)
Neck	0 (0)	Back kick	0 (0)	3rd round	4 (57.1)
Torso	0 (0)	Roundhouse kick	0 (0)	Overtime	0 (0)
Below waist	0 (0)	Side kick	0 (0)		
		360-spinning kick	0 (0)		
		Turning roundhouse kick	0 (0)		
		N/A	4 (57.1)		
		Mat	3 (42.9)		
Body location		Step		Injury time	
Anterior	4 (57.1)	Stand	6 (85.7)	00:00–00:30	1 (14.3)
Posterior	0 (0)	Forward	1 (14.3)	00:31–01:00	2 (28.6)
Lateral	3 (42.9)	Backward	0 (0)	01:01–01:30	1 (14.3)
		Side	0 (0)	01:31–02:00	3 (42.9)
Acceleration of head		Sparring stance		Location in court	
Sagittal	1 (14.3)	Open stance	2 (28.6)	1	0 (0)
Coronal	0 (0)	Closed stance	5 (71.4)	2	4 (57.1)
Transverse	3 (42.9)	N/A	0 (0)	3	3 (42.9)
Multiplane	3 (42.9)			Other	0 (0)

Results are expressed as numbers (*n*) and percentage (%). Results of tertiary contact are not presented as it was not observed.

## Data Availability

Not Applicable.
